# Periodic alternating nystagmus as a diagnostic clue in MRI-negative Wernicke encephalopathy mimicking multiple system atrophy: a case with complex heterogeneous dizziness

**DOI:** 10.3389/fneur.2026.1837183

**Published:** 2026-05-20

**Authors:** Hong Zhou, Ruiyun Zhang, Wei Wang

**Affiliations:** Department of Neurology, Civil Aviation General Hospital, Beijing, China

**Keywords:** dizziness, misdiagnosis, MRI-negative, orthostatic hypotension, periodic alternating nystagmus, Wernicke encephalopathy

## Abstract

Dizziness in elderly patients is heterogeneous and nonspecific, leading to frequent misdiagnosis. Wernicke encephalopathy (WE) is a severe but reversible nutritional disorder responsive to timely thiamine supplementation; it often lacks typical MRI changes and may mimic neurodegenerative diseases such as multiple system atrophy (MSA). A 62-year-old male with chronic heavy alcohol use presented with 1 year of recurrent heterogeneous dizziness, vertigo, unsteadiness, presyncope, falls, and nocturnal visual hallucinations. Severe orthostatic hypotension and pyramidal signs initially suggested MSA. Peripheral neuropathy was attributed to chronic alcohol intake. Brain MRI was normal without typical WE lesions. Videonystagmography revealed periodic alternating nystagmus (PAN), saccadic intrusions, and impaired smooth pursuit, indicating central vestibular dysfunction. These ocular abnormalities resolved rapidly after thiamine replacement, excluding MSA, confirming MRI-negative WE. With high-dose thiamine, cobalamin, and alcohol cessation, PAN disappeared, dizziness and hypotension improved markedly, and hallucinations resolved. Full functional recovery was achieved at 3-month follow-up. This case shows that WE may manifest with complex dizziness and normal MRI, mimicking MSA clinically. PAN is a key objective marker of central vestibular involvement. In alcoholic patients with unexplained dizziness, orthostatic intolerance and central ocular signs, reversible WE should be excluded before diagnosing progressive neurodegeneration.

## Introduction

Dizziness is one of the most common complaints in elderly patients, yet its etiological diagnosis remains challenging due to heterogeneous and fluctuating symptoms (1). Many patients are misdiagnosed with peripheral vertigo, cerebrovascular disease, cervical spondylosis, or neurodegenerative disorders (2).

Wernicke encephalopathy (WE) is an acute neuropsychiatric disorder caused by thiamine deficiency, most frequently associated with chronic alcohol abuse and malnutrition. Classic features include ophthalmoplegia, ataxia, and mental disturbance. However, atypical and MRI-negative presentations are common, leading to delayed or missed diagnosis (3, 4).

Periodic alternating nystagmus (PAN) is a rare spontaneous nystagmus characterized by periodic direction reversal, generally localizing to the cerebellum and brainstem (5, 6). Although PAN is well-recognized in neurodegenerative and vascular diseases (5, 7), its occurrence in WE with normal MRI has rarely been reported.

We describe a chronic heavy alcohol-using elderly patient with complex, heterogeneous dizziness that closely mimicked multiple system atrophy (MSA). In this case, PAN served as the key diagnostic clue leading to the diagnosis of MRI-negative WE. This case highlights the importance of promptly excluding treatable metabolic encephalopathies such as WE before diagnosing a progressive neurodegenerative disorder.

## Methods

A 62-year-old male was admitted to our hospital due to recurrent dizziness and falls for 1 year. The patient’s dizziness was heterogeneous and fluctuating in nature, including paroxysmal vertigo, postural disequilibrium, light-headedness, and presyncope triggered by orthostatic stress. He also reported nocturnal visual hallucinations, including seeing small animals, feeling mesh-like structures covering his body, and perceiving abnormal smoke-like images, which occurred independently of dizziness episodes. The patient had a 30-year history of heavy alcohol consumption, with a daily intake of approximately 250 mL of liquor, and had experienced significant weight loss of 25 kg within 2 years. His past medical history included hypertension, coronary artery disease, and old cerebral infarction.

Imaging examination included brain magnetic resonance imaging (MRI) to detect abnormal signal intensities in regions typically involved in Wernicke encephalopathy (mammillary bodies, thalamus, periaqueductal gray matter, brainstem), as well as cervical spinal cord MRI to evaluate for abnormal signals. Videonystagmography was conducted to evaluate eye movement function, including fixation and gaze holding test, smooth pursuit, saccadic movement, and spontaneous nystagmus detection. Laboratory testing for serum thiamine and serum vitamin B12 levels was also completed.

## Results

### Neurological examination results

Mild cognitive impairment was observed. The patient was alert and fully oriented during daytime admission, without obvious psychiatric disturbance or confusion. Visual hallucinations occurred only intermittently at night and were not present during the daytime examination. During cognitive assessment, he showed marked age-inappropriate prolonged response latency and slowed speech rate, independent of question difficulty. Orthostatic hypotension was confirmed: supine blood pressure was 149/81 mmHg with a heart rate of 82 beats per minute (bpm); standing blood pressure decreased to 108/50 mmHg with a heart rate of 69 bpm. Eye movements were full in all directions without significant nystagmus. Limb coordination was normal; trunk ataxia could not be fully evaluated due to persistent dizziness. Hyperactive tendon reflexes, increased muscle tone were present in all four limbs, and decreased pain sensation and proprioception below both knees.

### Investigation results

**Figure 1:**
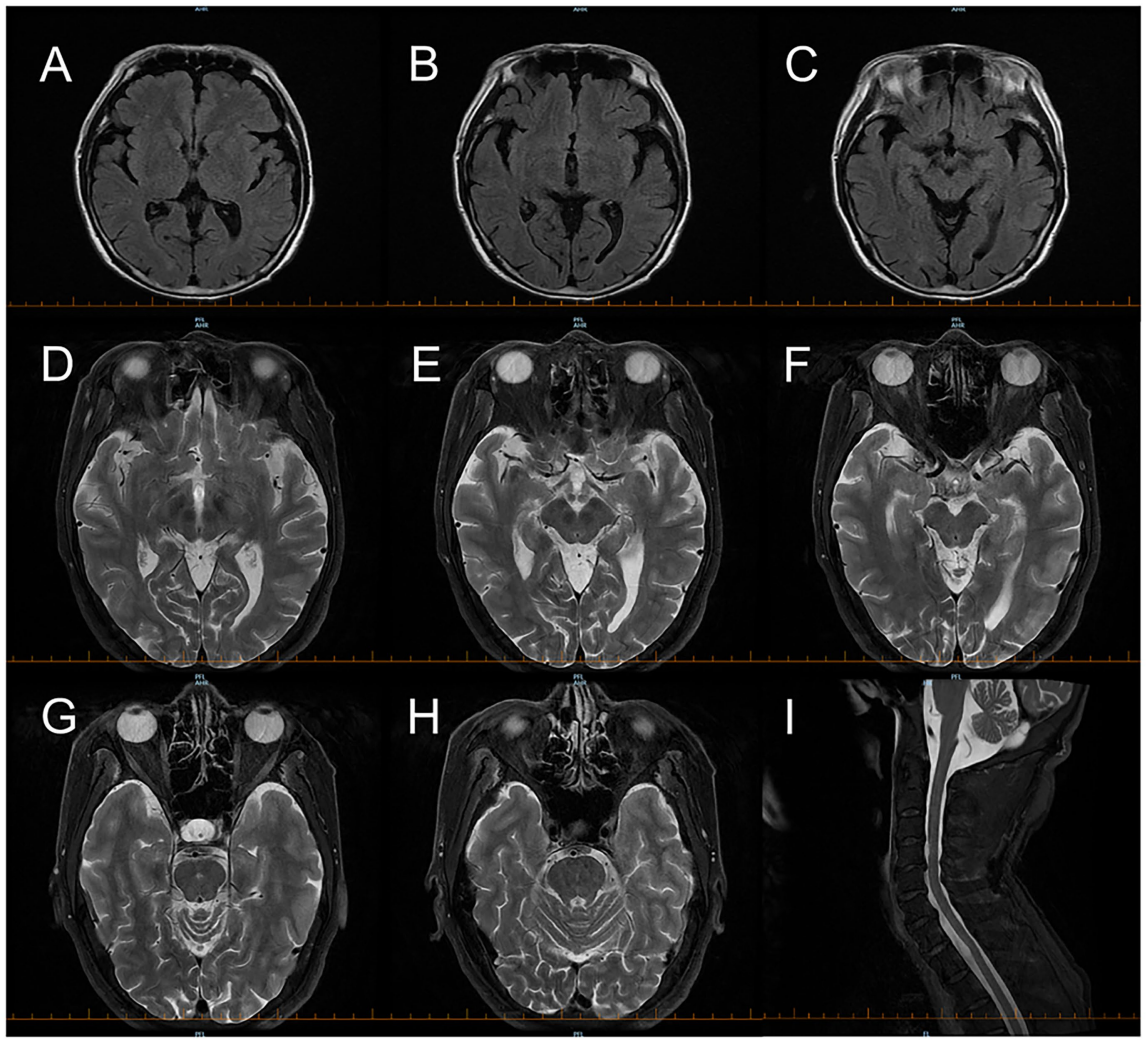
Brain MRI of the 62-year-old male patient demonstrates no typical features of Wernicke encephalopathy. **(A–C)** Axial FLAIR sequence: No abnormal hyperintensities are seen in the mammillary bodies, thalamus, periaqueductal gray matter, or brainstem. **(D–H)** Follow-up thin-section axial T2-weighted skull base images show no definite abnormal signals. Cervical spinal cord MRI **(I)** (T2-weighted) shows no characteristic lesions of subacute combined degeneration.

**Figure 2:**
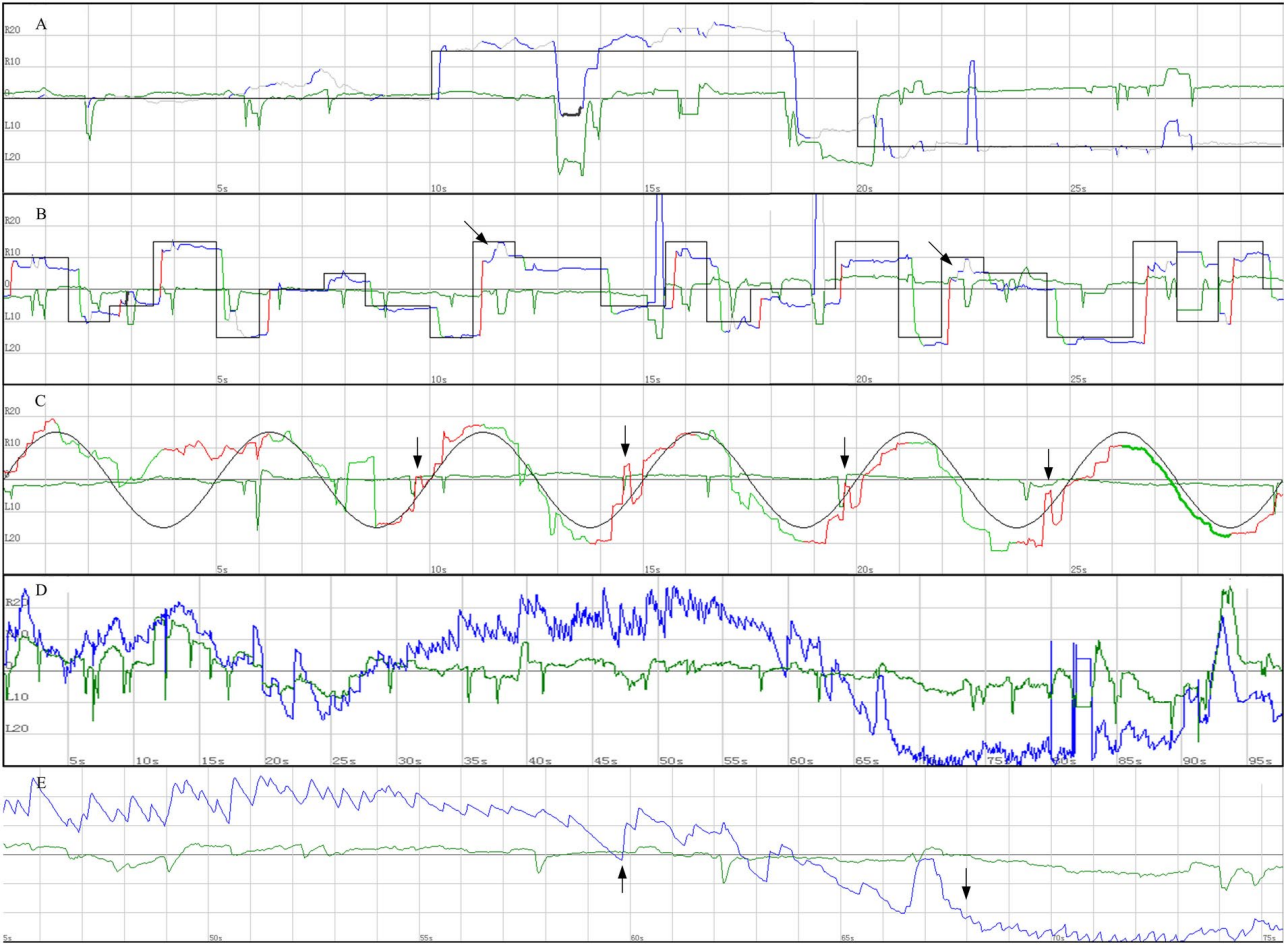
Videonystagmography findings of the 62-year-old male patient. **(A)** Fixation and gaze holding test. **(B)** Reflexive saccade test showing saccadic hypometria (indicated by arrows). **(C)** Smooth pursuit test showing saccade intrusions (indicated by arrows). **(D)** Spontaneous compressed tracing demonstrating a full cycle of periodic alternating nystagmus (PAN) within 100 s. **(E)** Magnified tracing from 45 to 75 s in panel D, showing detailed changes in eye position. The upward arrow indicates gradually attenuated right-beating nystagmus, and the downward arrow indicates gradually increased left-beating nystagmus. **(A–C)** The black line indicates the target reference; colored traces show the actual eye movement. **(D,E)** The blue trace denotes horizontal eye movement and the green trace denotes vertical eye movement. R, right; L, left. Bilateral eye movements were recorded simultaneously, and only the left eye traces are presented here.

Brain MRI showed no abnormal signal intensities in the mammillary bodies, thalamus, periaqueductal gray matter, or brainstem—regions typically involved in WE (Figure 1). Cervical spinal cord MRI was normal, with no findings suggestive of subacute combined degeneration (Figure 1). Videonystagmography revealed spontaneous periodic alternating nystagmus (PAN), accompanied by impaired smooth pursuit and saccadic hypometria (Figure 2), confirming central vestibular dysfunction. To further demonstrate the dynamic characteristics of periodic alternating nystagmus, Supplementary Video S1 is provided. Laboratory investigations revealed a serum thiamine level <1.0 ng/mL (lower limit of normal: 1.0 ng/mL), and a serum vitamin B12 level of 111 pmol/L (normal ≥133 pmol/L).

### Treatment and follow-up results

The patient was administered intravenous thiamine 300 mg daily and intramuscular cobalamin 1.5 mg daily for vitamin replacement. Symptomatic treatment included midodrine for orthostatic hypotension, baclofen for nystagmus, and oxazepam for alcohol withdrawal syndrome. Complete alcohol cessation was strongly advised and successfully achieved.

After 11 days of treatment, PAN resolved completely. Orthostatic hypotension improved markedly at 15 days post-treatment. At the 1-month follow-up, the patient’s dizziness was significantly alleviated, animal-related visual hallucinations had disappeared, and only mild residual mesh-like sensations persisted. At the 2-month follow-up, dizziness further improved, and the patient was able to perform daily activities independently. At the 3-month follow-up, dizziness and visual hallucinations resolved completely, with full functional recovery.

## Discussion

This case highlights four key clinical points with important implications for clinical practice, which are discussed in detail as follows:

### Heterogeneous dizziness can obscure the underlying etiology

The patient’s dizziness presented with a heterogeneous spectrum of symptoms including vertigo, postural imbalance, and presyncope—a common yet nonspecific clinical presentation in elderly patients. Such fluctuating, multifaceted symptom profiles often lead to clinical confusion, delayed etiological diagnosis, and misdirection of further workup, as clinicians frequently attribute these vague symptoms to benign peripheral vertigo, cervical spondylosis or age-related cerebrovascular changes. However, the presence of central oculomotor signs in this patient provided a critical diagnostic clue that redirected investigation toward central neurological pathology, rather than peripheral or benign causes.

### PAN indicates central vestibular involvement

PAN is a distinct central oculomotor sign, typically associated with structural or functional lesions of the cerebellum and brainstem, particularly the vestibulocerebellar regions including the nodulus and uvula (5, 6). While PAN has been rarely reported in peripheral vestibular disorders, it is most consistent with central pathology, especially when accompanied by other central oculomotor abnormalities. In this patient, the coexistence of PAN, saccadic intrusion, and impaired smooth pursuit on videonystagmography confirmed central vestibular system dysfunction, despite the complete absence of abnormal findings on brain MRI. This finding underscores the indispensable value of targeted oculomotor testing in identifying central pathology, even when routine neuroimaging is unremarkable.

### Nutritional deficiency related to alcohol abuse contributes to orthostatic hypotension

The patient had a long history of alcohol abuse and significant weight loss, both major risk factors for thiamine deficiency and WE. Instead, this patient did not exhibit the classic clinical trial of WE, but demonstrated atypical clinical features consistent with alcohol-related WE (3). Neurological symptoms improved significantly after thiamine supplementation, supporting the diagnosis of atypical WE. However, the severe orthostatic hypotension observed in this patient could not be fully explained by thiamine deficiency alone.

Laboratory investigations revealed a serum vitamin B12 level of 111 pmol/L, which was below the lower limit of the normal reference range (133 pmol/L), confirming the presence of vitamin B12 deficiency. Previous studies have demonstrated that vitamin B12 deficiency can damage the autonomic nervous system, leading to impaired sympathetic nerve function, insufficient norepinephrine release, and abnormal baroreflex regulation—all of which are important mechanisms underlying the development of orthostatic hypotension (8, 9). Importantly, autonomic dysfunction may occur as an isolated or prominent clinical manifestation of vitamin B12 deficiency, even in the absence of severe megaloblastic anemia or subacute combined degeneration of the spinal cord (10).

Patients with chronic alcoholism frequently develop combined deficiencies of thiamine and vitamin B12 due to inadequate intake and malabsorption (11, 12). The coexistence of these two deficiencies can lead to overlapping clinical features, increasing the diagnostic complexity. In this case, orthostatic hypotension markedly improved after vitamin B12 replacement, further supporting that vitamin B12 deficiency-related autonomic neuropathy was the underlying cause of the patient’s orthostatic hypotension (10).

Nevertheless, previous studies have reported that vitamin B12 deficiency can also cause dizziness, central ocular motor abnormalities, and neuropsychiatric disturbances (13–15). Although both cranial and cervical spinal cord MRI were unremarkable in this patient, clinical improvements were observed after vitamin B12 supplementation. Therefore, the superimposed influence of vitamin B12 deficiency on the patient’s dizziness, psychiatric symptoms and oculomotor dysfunction cannot be ruled out.

Therefore, in patients with alcohol-related WE who present with orthostatic hypotension not fully explained by WE itself, routine screening for vitamin B12 deficiency is strongly recommended. Early identification and correction of dual vitamin deficiencies may significantly improve autonomic nervous system function and overall clinical outcomes (16, 17).

### Atypical MRI-negative WE with orthostatic hypotension can mimic MSA

This patient exhibited multiple clinical features highly suggestive of MSA, including chronic progressive dizziness, severe orthostatic hypotension, pyramidal tract signs, central nystagmus, and normal brain MRI findings (18). However, a long history of heavy alcohol use, weight loss, significant malnutrition, nocturnal visual hallucinations and peripheral neuropathy, were not consistent with MSA and instead favored an alternative diagnosis of atypical alcohol-related WE. Notably, up to 40% of patients with acute or mild WE may have normal brain MRI (4), so a normal MRI does not exclude the diagnosis of WE. This case illustrates that atypical, MRI-negative WE should be considered in the differential diagnosis, especially when it presents with prominent autonomic dysfunction such as orthostatic hypotension and mimics MSA.

### Clinical implications

For elderly patients with a history of alcohol misuse, unexplained weight loss, visual hallucinations, or central oculomotor signs accompanied by unexplained dizziness, empirical thiamine supplementation should be initiated immediately to avoid irreversible neurological damage caused by delayed treatment of WE. In clinical practice, treatable metabolic encephalopathies such as WE must be thoroughly excluded before a diagnosis of progressive neurodegenerative disorders such as MSA is made. This approach is crucial for improving patient prognosis and avoiding unnecessary interventions.

This case confirms that atypical WE may present with complex heterogeneous dizziness and normal brain MRI findings, and is prone to misdiagnosis as MSA in clinical settings. PAN is a reliable objective neurological sign indicative of central vestibular involvement, which can facilitate the early diagnosis of MRI-negative WE. Unlike MSA, this atypical form of WE is completely reversible with timely and adequate thiamine and vitamin B12 replacement therapy combined with alcohol cessation. For elderly patients with unexplained dizziness, orthostatic hypotension, and central oculomotor abnormalities, clinicians should prioritize the consideration of treatable metabolic encephalopathies such as WE in the differential diagnosis process to reduce diagnostic errors and improve patient outcomes.

## Data Availability

The original contributions presented in the study are included in the article/Supplementary material, further inquiries can be directed to the corresponding author.

## Supplementary material

Supplementary Video S1 Spontaneous eye movement recording over 3 min in this 62-year-old male patient, showing repeated alternating phases of periodic alternating nystagmus. Real-time eye records and simultaneous eye movement recordings are presented. The red line indicates horizontal eye movement, and the green line indicates vertical eye movement. Notably, a vertical component was observed during approximately 113–128 s.
